# Parametric Control via the Algebraic Expression of Lotus-Type Pore Shapes in Metals

**DOI:** 10.3390/ma17123013

**Published:** 2024-06-19

**Authors:** Liwei Wang, Bo-Yue Lee, Peng-Sheng Wei, Mingming Quan

**Affiliations:** 1 School of Material Science and Engineering, Hebei University of Science and Technology, Shijiazhuang 050018, China; wangliwei110127@163.com (L.W.); qmm1999411@163.com (M.Q.); 2 Department of Mechanical and Electro-Mechanical Engineering, National Sun Yat-sen University, Kaohsiung 80424, Taiwan; yuebo997@gmail.com

**Keywords:** porosity, lotus-type pore shape, solidification, bubble entrapment, Sieverts’ law

## Abstract

Lotus-type porous metals, characterized by low densities, large surface areas, and directional properties, are contemporarily utilized as lightweight, catalytic, and energy-damping materials; heat sinks; etc. In this study, the effects of dimensionless working parameters on the morphology of lotus-type pores in metals during unidirectional solidification were extensively investigated via general algebraic expressions. The independent dimensionless parameters include metallurgical, transport, and geometrical parameters such as Sieverts’ law constant, a partition coefficient, the solidification rate, a mass transfer coefficient, the imposed mole fraction of a solute gas, the total pressure at the top free surface, hydrostatic pressure, a solute transport parameter, inter-pore spacing, and initial contact angle. This model accounts for transient gas pressure in the pore, affected by the solute transfer, gas, capillary, and hydrostatic pressures, and Sieverts’ laws at the bubble cap and top free surface. Solute transport across the cap accounts for solute convection at the cap and the amount of solute rejected by the solidification front into the pore. The shape of lotus-type pores can be described using a proposed fifth-degree polynomial approximation, which captures the major portions between the initial contact angle and the maximum radius at a contact angle of 90 degrees, obtained by conserving the total solute content in the system. The proposed polynomial approximation, along with its working parameters, offers profound insights into the formation and shape of lotus-type pores in metals. It systematically provides deep insights into mechanisms that may not be easily revealed with experimental studies. The prediction of a lotus-type pore shape is thus algebraically achieved in good agreement with the available experimental data and previous analytical results.

## 1. Introduction

Porous materials such as lotus-type porous metals feature good direction-dependent mechanical properties, such as tensile, compression, bending, and fatigue strength, and physical and thermal properties, such as sound absorption and thermal and electrical conductivity [1,2,3,4,5,6]. These properties act better in parallel than perpendicularly [1]. Lotus-type porous materials can thus be functionally utilized in various biomedical, micro-, and nano-technologies [1]. For example, in view of direction-dependent Young’s modulus and lightweight, lotus-type porous magnesium has been used for scaffolds in tissue engineering. Lotus-type porous magnesium with excellent biocompatibility, biodegradability, and bioresorbability [7] is also becoming a promising material used for the formation of artificial bones in bioengineering. Lotus-type porous magnesium permits good attachment of tissues to a surface, promotes cell ingrowth, and inhibits bacterial adhesion. Moreover, lotus-type porous stainless steel and titanium have been used for artificial bones and dental implants, showing good corrosion resistance, light weights, and high strength. Furthermore, the lotus-type porous structures in micro- and nano-technologies exhibit good cooling performance due to the fluid flow along the direction of the pores having smaller pressure drops and higher effective thermal conductivities and are thus considered a special kind of micro-channel structure [1,8,9]. The pore size, direction, and porosity of lotus-type porous metals can be controlled by changing the imposed solute gas pressure during melting and solidification, the solidification velocity, and the temperature gradient. This becomes the aim of the parametric and extensive study of the pore shape development during the so-called gasarite solidification, similar to the unidirectional transformation of eutectics from liquid to two different solids in this work.

The shapes of lotus-type porous metals can be systematically and rigorously understood using theoretical studies. Based on the analytical solute concentration in liquids and the minimum undercooling criterion provided by the Jackson–Hunt model during eutectic solidification, Liu et al. [10] predicted and experimentally confirmed decreases in porosity, pore radius, and inter-pore spacing as solidification speed, total gas pressure, and partial pressure of hydrogen and argon increased during the gasarite solidification of copper. The Jackson–Hunt theory and analytical solutions of solute concentration were, however, criticized by Lee et al. [11], Drenchev et al. [12], and Wei et al. [13]. Lu et al. [14] also provided a simple thermodynamic model to describe the relationship between processing variables and the pore structure for the ordered porosity copper fabricated via directional solidification. The deviation between the calculated and experimental values was attributed to hydrogen diffusion in liquid metal. A rather general analytical solute concentration on the gasar eutectic solidification of, for example, copper-containing hydrogen, was provided by Li et al. [15] using a multiple scale expansion and matching method. The interfacial boundary conditions were that the interface temperature at the solidification front was satisfied by the Gibbs–Thomson condition, the solute gas pressure at the gas–liquid interface by Sieverts’ law, and the balance of surface tensions at the three-phase line by Young’s relation. Involving complicated products and the divisions of Bessel functions, the predicted results showed that the porosity and height of the bubble cap decreased as the pressures of the dissolving and non-dissolving gases increased. It also showed that an increase in solidification rate decreased porosity.

An unsteady three-dimensional concentration equation during gasarite solidification was numerically solved by Drenchev et al. [16], finding that an increase in solidification rate enhanced solute transfer to either the pore cap or the top of the liquid. Yang et al. [17] also showed that solute transport along the liquid–solid interface into the cap increases with the solidification rate. As the solidification rate increases, solute transport can be enhanced, and the direction can be changed from the bottom to the top of the liquid. Iitsuka et al. [18] used the multi-phase field method to solve pore growth during the gasarite solidification of aluminum. Introducing a mass transfer coefficient to avoid complicated computations of the concentration field, Wei et al. [19] proposed that, as the thickness of the concentration boundary layer is much less than the height of the bubble cap, a solute gas is transferred from the bubble to the liquid in the early stages. On the other hand, solute transport can occur from the surrounding liquid into the pore for a thick concentration boundary layer on the solidification front. Combining these two cases, Wei and Lee [20] provided a unified algebraic expression for the shapes of lotus-type pores.

In this study, the effects of dimensionless working parameters on lotus-type pore shapes during the unidirectional solidification of metals are thoroughly predicted via algebraic expressions. The shape of the lotus-type pores is delineated in a closed form, which requires determining the length and maximum radius at a contact angle of 90 degrees [20], using the integral method to approximate solute concentration profiles in the boundary layers on advancing liquid–solid interfaces and bubble caps. Gas–liquid interfaces are satisfied by the Young–Laplace equation and Sieverts’ law. Our results successfully control the formation, shape, and porosity of lotus-type pores in metals in advance.

## 2. Algebraic Integral Prediction

**Figure 1:**
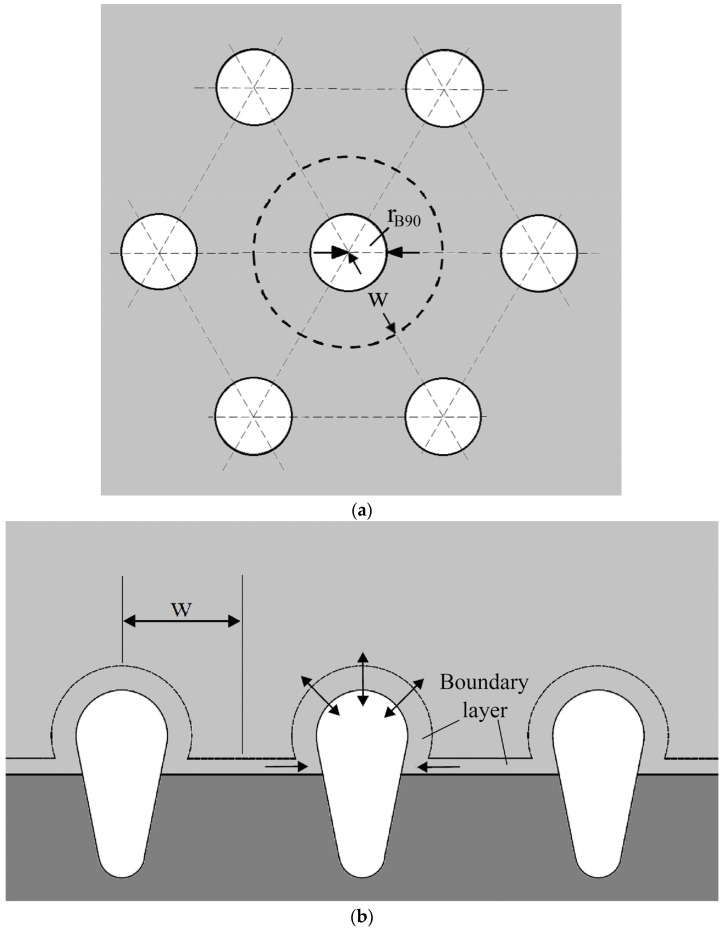
Sketch of lotus-type pores with (**a**) transverse and (**b**) longitudinal cross-sections [20].

**Figure 2:**
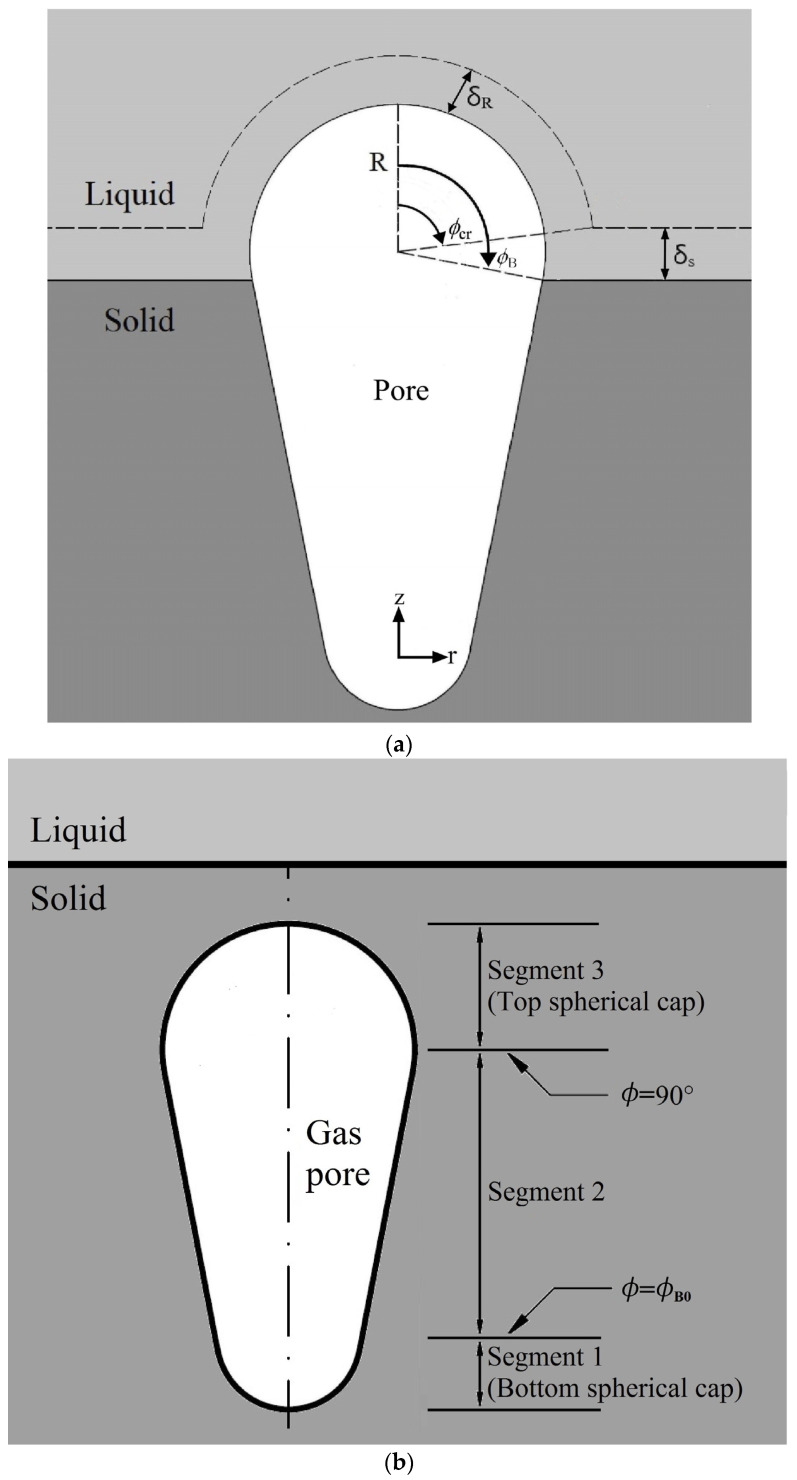
(**a**) Solute transport model and (**b**) segments for analysis [20].

Lotus-type pores with transverse and longitudinal cross-sections are sketched in Figure 1a,b, respectively, based on a model in a previous study [20]. The development of lotus-type pores, governed by its internal solute gas pressure, results from solute transfer from the concentration boundary layers on the cap and on the advancing liquid–solid interface, as illustrated in Figure 2a. The bottom and top of tiny pores can be spherical in shape, with contact angles greater than the initial angle and less than 90 degrees, respectively, as illustrated in Figure 2b [20]. Other assumptions can be referenced from a previous work [20]. The major portion, namely the second segment between the initial contact angle and the maximum radius at a contact angle of 90 degrees, is delineated as follows:

### 2.1. Shape of the Second Segment

The shape of the second segment can be predicted using polynomials of different degrees [19], as will be shown later. Here, we show that the second segment can be accurately and reliably described using a polynomial with five degrees [19]:

(1) $$r=a+b(\frac{s}{l_{90}})+c{\left(\frac{s}{l_{90}}\right)}^{2}+d{\left(\frac{s}{l_{90}}\right)}^{3}+e{\left(\frac{s}{l_{90}}\right)}^{4}+f{\left(\frac{s}{l_{90}}\right)}^{5}\;\mathrm{for}\;90^{0}\leq \phi \leq \phi_{B0}$$

where *s* and $l_{90}$ are, respectively, the solidification front location and length of the second segment. The coefficients in Equation (1) are determined by substituting the following conditions: *r* = $sin\phi_{B0}$, *dr*/*ds* = −1/$tan\phi_{B0}$ at *s* = 0, and *r* = $r_{B90}$, *dr*/*ds* = $d^{2}r/ds^{2}$ = $d^{3}r/ds^{3}$ = 0 at *s* = $l_{90}$. They are thus given by the following:

(2) $$a=sin\phi_{B0}$$

(3) $$b=-\frac{l_{90}}{tan\phi_{B0}}$$

(4) $$c=10(r_{B90}-sin\phi_{B0})+\frac{4l_{90}}{tan\phi_{B0}}$$

(5) $$d=-2c+\frac{2l_{90}}{tan\phi_{B0}}=-20(r_{B90}-sin\phi_{B0})-\frac{6l_{90}}{tan\phi_{B0}}$$

(6) $$e=-\frac{c}{2}-d=15(r_{B90}-sin\phi_{B0})+\frac{4l_{90}}{tan\phi_{B0}}$$

(7) $$f=-\frac{d}{10}-\frac{2e}{5}=-4(r_{B90}-sin\phi_{B0})-\frac{l_{90}}{tan\phi_{B0}}$$

where the dimensionless length and maximum radius of the second segment can be found as follows [20].

### 2.2. Dimensionless Length of the Second Segment

The sum of solutes in the pore and the concentration boundary layers on the cap and on the advancing solid–liquid interface is conserved, as given by the following [20]:

(8) $$\begin{array}{l} \rho_{g}V+\int_{R}^{R+\delta_{R}}\left(C_{l}-C_{\infty }\right)2\pi r^{2}(1-cos\phi_{cr})dr+\int_{s}^{s+\delta_{s}}\left(C_{l}-C_{\infty })\pi (w^{2}-R^{2}{sin}^{2}\phi_{B}\right)dz\\ \;=const.\\ \;=\rho_{g0}V_{0}+\int_{R_{0}}^{R_{0}+\delta_{R0}}\left(C_{l}-C_{\infty }\right)2\pi r^{2}(1-cos\phi_{cr0})dr+\int_{0}^{\delta_{s0}}\left(C_{l}-C_{\infty }\right)\pi (w^{2}-R_{0}^{2}{sin}^{2}\phi_{B0})dz \end{array}$$

The integral method is used to evaluate the integrals in Equation (8) by substituting binomial expressions of the concentration profiles, leading to the dimensionless length of the second segment:

(9) $$\begin{array}{cc} l_{90}= & \frac{1}{(\frac{2}{3}r_{B90}^{2}+\frac{1}{3}{sin}^{2}\phi_{B0})p_{g90}}\{\frac{4p_{g0}}{3}-p_{g90}F_{3}(\phi_{B0},r_{B90})\\ & +(\frac{\sqrt{p_{g0}}}{K_{Sc}}-\frac{\sqrt{\gamma p_{a}}}{K_{S\infty }})F_{1}(\phi_{B0},h_{D0},U_{0})-(\frac{\sqrt{p_{g90}}}{K_{Sc}}-\frac{\sqrt{\gamma p_{a}}}{K_{S\infty }})F_{2}(r_{B90},h_{D90},U_{90})\\ & +\frac{\sqrt{\gamma p_{a}}}{K_{S\infty }}F_{4}(\phi_{B0},k_{p0},r_{B0},w,U_{0})-\frac{\sqrt{\gamma p_{a}}}{K_{S\infty }}F_{5}(k_{p^{90}},r_{B90},w,U_{90})\} \end{array}$$

where the coefficient functions are as follows:

(10) $$F_{1}(\phi_{B0},h_{D0},U_{0})\equiv \frac{2}{30}(1-\frac{\frac{2}{U_{0}}+cos\phi_{B0}}{1+\frac{2}{h_{D0}}})[{\left(\frac{2}{h_{D0}}\right)}^{3}+5{\left(\frac{2}{h_{D0}}\right)}^{2}+10\frac{2}{h_{D0}}]$$

(11) $$F_{2}(r_{B90},h_{D90},U_{90})\equiv \frac{2r_{B90}^{3}}{30}(1-\frac{\frac{2}{U_{90}r_{B90}}}{1+\frac{2}{h_{D90}r_{B90}}})[{\left(\frac{2}{h_{D90}r_{B90}}\right)}^{3}+5{\left(\frac{2}{h_{D90}r_{B90}}\right)}^{2}+10\frac{2}{h_{D90}r_{B90}}]$$

(12) $$F_{3}(\phi_{B0},r_{B90})\equiv \frac{2r_{B90}^{3}}{3}+\frac{1}{3}(2-cos\phi_{B0}){\left(1+cos\phi_{B0}\right)}^{2}$$

(13) $$F_{4}(\phi_{B0},k_{p0},r_{B0},w,U_{0})\equiv (\frac{1}{k_{p0}}-1)(w^{2}-{sin}^{2}\phi_{B0})\frac{2}{3U_{0}}$$

(14) $$F_{5}(k_{p90},r_{B90},w,U_{90})\equiv (\frac{1}{k_{p90}}-1)(w^{2}-r_{B90}^{2})\frac{2}{3U_{90}}$$

Functions $F_{1}$, $F_{2}$, $F_{4}$, and $F_{5}$, defined by Equations (10), (11), (13) and (14), are related to the thicknesses of the concentration boundary layers on the cap and solidification front at the initial time and at the time corresponding to the maximum radius, respectively, whereas function $F_{3}$ in Equation (12) refers to the sum of the volumes of the top and bottom caps. Sieverts’ law imposed on the cap and top surface are, respectively, as follows:

(15) $$C_{c}=\frac{\sqrt{p_{g}}}{K_{Sc}Bo},\;C_{\infty }=\frac{\sqrt{\gamma p_{a}}}{K_{S\infty }Bo}$$

The dimensionless solute gas pressure in the pore at the initial time and at the maximum radius can be evaluated by applying the Young–Laplace equation to the cap:

(16) $$p_{g0}=Bo(h_{B0}-z_{B0})+p_{a}+2$$

(17) $$p_{g90}=p_{g0}+\frac{2}{r_{B90}}-2$$

### 2.3. Dimensionless Maximum Radius

The dimensional unsteady solute content in the pore yields the following:

(18) $$\frac{d{\tilde{\rho }}_{g}\tilde{V}}{d\tilde{t}}=A_{cr}{\tilde{h}}_{D}({\tilde{C}}_{\infty }-{\tilde{C}}_{c})+\pi \eta ({\tilde{w}}^{2}-{\tilde{R}}^{2}{sin}^{2}\phi_{B})\tilde{U}{\tilde{C}}_{\infty }(\frac{1}{k_{p}}-1)$$

where terms on the right hand represent solute convection through the bubble cap and the amount of solute rejected by the solidification front, and the term on the left-hand side reduces to $d{\tilde{\rho }}_{g}\tilde{V}/d\tilde{t}=({\tilde{p}}_{g90}/{\tilde{R}}_{u}{\tilde{T}}_{m})\pi {\tilde{r}}_{B90}^{2}\tilde{U}$ at the maximum radius. The dimensionless form of Equation (18) at the maximum radius thus becomes

(19) $$\frac{1}{Bo}p_{g90}\pi r_{B90}^{2}U=A_{cr}h_{D}(C_{\infty }-C_{c})+\pi \eta (w^{2}-R^{2}{sin}^{2}\phi_{B})UC_{\infty }(\frac{1}{k_{p}}-1)$$

Equation (19) gives the dimensionless maximum radius [20]

(20) $$A_{S}r_{B90}^{6}+B_{S}r_{B90}^{5}+C_{S}r_{B90}^{4}+D_{S}r_{B90}^{3}+E_{S}r_{B90}^{2}+F_{S}r_{B90}+G_{S}=0$$

with

(21) $$A_{S}=a_{S}^{2}-{\left(\frac{2h_{D90}}{K_{S}U_{90}}\right)}^{2}[Bo(h_{B0}-z_{B0})+p_{a}]$$

(22) $$B_{S}={\overset{⌢}{B}}_{S}+\frac{4A_{S}}{h_{D90}}$$

(23) $$C_{S}={\overset{⌢}{C}}_{S}+\frac{2}{h_{D90}}(\frac{2A_{S}}{h_{D90}}+{\overset{⌢}{B}}_{S}-c_{S})$$

(24) $$D_{S}={\overset{⌢}{D}}_{S}+\frac{2}{h_{D90}}[4a_{S}\sqrt{{\overset{⌢}{E}}_{S}}+4b_{S}+\frac{2}{U_{90}}{\left(\frac{4h_{D90}}{K_{S}U_{90}}\right)}^{2}-\frac{2c_{S}}{h_{D90}}]$$

(25) $$E_{S}={\overset{⌢}{E}}_{S}+\frac{2}{h_{D90}}[\frac{8}{h_{D90}}+(\frac{4a_{S}}{h_{D90}}-4-2b_{S})\sqrt{{\overset{⌢}{E}}_{S}}]$$

(26) $$F_{S}=\frac{2}{h_{D90}}(2{\overset{⌢}{E}}_{S}-\frac{8}{h_{D90}}\sqrt{{\overset{⌢}{E}}_{S}})$$

(27) $$G_{S}={\left(\frac{2}{h_{D90}}\right)}^{2}{\overset{⌢}{E}}_{S}$$

(28) $$a_{S}\equiv -Bo(h_{B0}-z_{B0})-p_{a}-\eta \frac{\sqrt{\gamma p_{a}}}{K_{S}}(\frac{1}{k_{p90}}-1)+\frac{2h_{D90}}{K_{S}U_{90}}\sqrt{\gamma p_{a}}$$

(29) $$b_{S}\equiv 2+\frac{2}{U_{90}}\frac{2h_{D90}}{U_{90}K_{S}}\sqrt{\gamma p_{a}}$$

(30) $$c_{S}=2{\left(\frac{2h_{D90}}{K_{S}U_{90}}\right)}^{2}+4a_{S}$$

and

(31) $${\overset{⌢}{B}}_{S}=-2a_{S}b_{S}-2{\left(\frac{2h_{D90}}{K_{S}U_{90}}\right)}^{2}+\frac{4(a_{S}^{2}-A_{S})}{U_{90}}$$

(32) $${\overset{⌢}{C}}_{S}=b_{S}^{2}+\frac{8}{U_{90}}{\left(\frac{2h_{D90}}{U_{90}K_{S}}\right)}^{2}+{\left(\frac{2}{U_{90}}\right)}^{2}(A_{S}-a_{S}^{2})+2a_{S}\sqrt{{\overset{⌢}{E}}_{S}}$$

(33) $${\overset{⌢}{D}}_{S}=-2b_{S}\sqrt{{\overset{⌢}{E}}_{S}}-2{\left(\frac{2}{U_{90}}\frac{2h_{D90}}{K_{S}U_{90}}\right)}^{2}$$

(34) $${\overset{⌢}{E}}_{S}=\eta^{2}w^{4}\frac{\gamma p_{a}}{K_{S}^{2}}{\left(\frac{1}{k_{p90}}-1\right)}^{2}$$

Algebraic Equation (20), together with the coefficients from Equations (21)–(34), can be solved using Microsoft Excel 2010 based on the Generalized Reduced Gradient (GRG) solver, which is a nonlinear optimization code considered to be one of the most robust and reliable approaches for solving difficult nonlinear programming problems. The iteration number was 100, and the relative error was 0.001.

## 3. Results and Discussion

The present work constitutes a dimensionless study that is readily accessible and applicable across a wide range of applications [21]. It offers a comprehensive analysis of various dimensionless operating parameters on the shapes of lotus pores. In this study, the shapes of lotus-type pores were investigated by taking into account the solute amount in the pore, the boundary layers on the cap, and the advancing liquid–solid interface, affected by different dimensionless working parameters, including Sieverts’ law constant, the solidification rate, a partition coefficient, a mass transfer coefficient, the imposed solute gas concentration and ambient pressure at the top surface, hydrostatic pressure, the solute transport parameter, the initial contact angle, and inter-pore spacing. Since the solubility of the gas atoms in the molten metal plays a major role in the formation of pores, the algebraic equation accounts for the solubility of gases in liquid metals by imposing Sieverts’ law at the bubble cap and top free surface. The length of the lotus-type pores or the pore length is approximated by the length between the initial contact angle and the maximum radius, given by Equation (9) together with the function coefficients of Equations (10)–(14), the interfacial concentration from Sieverts’ law in Equation (15), and the balance of pressures from the Young–Laplace equations in Equations (16) and (17) at the cap and top free surface and at different times. The maximum radius is determined using Equation (20) together with the coefficient functions in Equations (21)–(34). The shape of lotus-type pores therefore depends on the solute amount in the boundary layer on the cap, governed by $\left(\sqrt{p_{g0}}/K_{Sc}-\sqrt{\gamma p_{a}}/K_{S\infty }\right)$$F_{1}(\phi_{B0},h_{D0},U_{0})$ and $\left(\sqrt{p_{g90}}/K_{Sc}-\sqrt{\gamma p_{a}}/K_{S\infty }\right)$$F_{2}(r_{B90},h_{D90},U_{90})$, and on the solidification front from ($\sqrt{\gamma p_{a}}/K_{S\infty }$)$F_{4}(\phi_{B0},k_{p0},r_{B0},w,U_{0})$ and ($\sqrt{\gamma p_{a}}/K_{S\infty }$)$F_{5}(k_{p90},r_{B90},w,U_{90})$ at the initial time and at the maximum radius, respectively, as well as on the solute amount in the top and bottom spherical caps at the maximum radius, $p_{g90}$$F_{3}(\phi_{B0},r_{B90})$. The thicknesses of the concentration boundary layer on the cap at the initial time and the maximum radius, related to functions $F_{1}(\phi_{B0},h_{D0},U_{0})$ and $F_{2}(r_{B90},h_{D90},U_{90})$ (see Equations (10) and (11)), increase as the dimensionless mass transfer coefficient decreases, whereas those on the solidification front, governed by functions $F_{4}(\phi_{B0},k_{p0},r_{B0},w,U_{0})$ and $F_{5}(k_{p90},r_{B90},w,U_{90})$ (see Equations (13) and (14)), increase as solidification rate decreases.

The typical values of the dimensionless parameters in this work are $K_{Sc}$ = $K_{S\infty }$ = 2, $U_{0}$ = 0.8, $U_{90}$ = 0.4, $k_{p0}$ = 0.002, $k_{p90}$ = 0.0018, $h_{D0}$ = 0.1, $h_{D90}$ = 0.05, γ = 1, $p_{a}$ = 790, $Bo(h_{B0}-z_{B0})$ = 30, $\phi_{B0}$ = 140°, w = 2.5, and η = 1. The solidification rate usually decreases during solidification. The scaling of the mass transfer coefficient on the cap ${\tilde{h}}_{D}\approx {\tilde{D}}_{l}/{\tilde{\delta }}_{s}$ thus decreases during the course of solidification because of an increase in the concentration boundary layer thickness on the advancing liquid–solid interface given by ${\tilde{\delta }}_{s}\approx 2{\tilde{D}}_{l}/\tilde{U}$ [20]. In view of this decrease with solidification rate, the partition coefficient also decreases during solidification. Physically speaking, the partition coefficient representing the ratio between the solute concentration in a solid to that in a liquid at the solidification front can be affected by fluid convection, the solvent and solute, and the characteristics of the solidification front. The solute transport parameter η takes into account the partial transport of the solutes rejected by the solidification front into the bubble.

**Figure 3:**
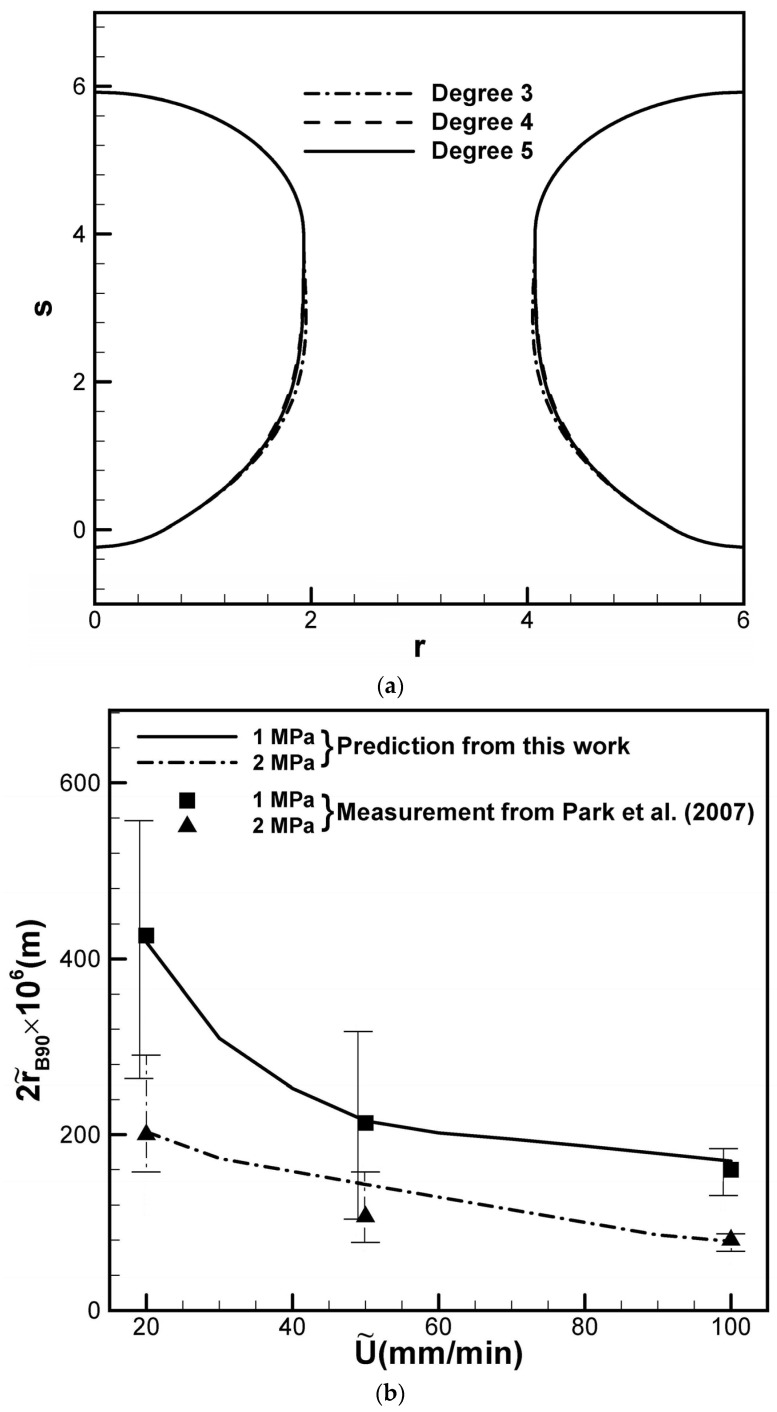

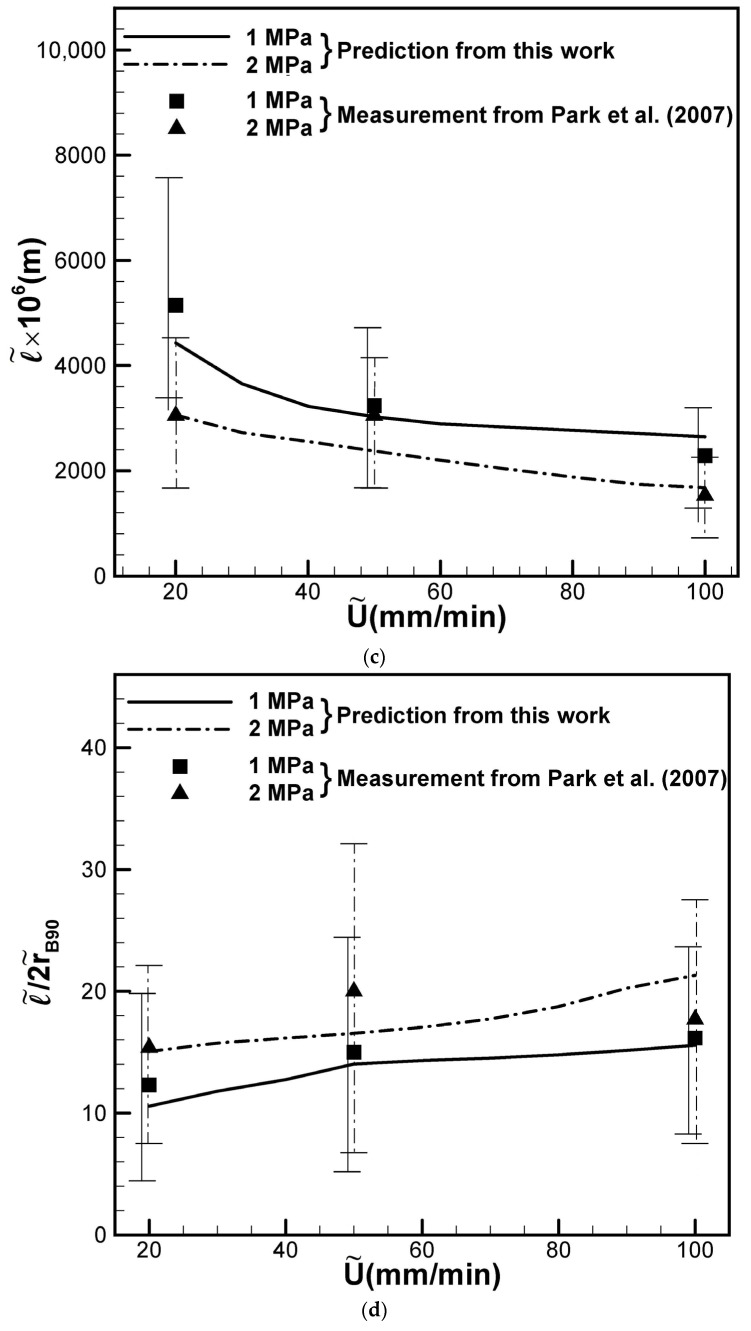
Comparisons of (**a**) polynomials of different degrees, and prediction and measurement of (**b**) diameter, (**c**) length, and (**d**) aspect ratio versus solidification rate for different imposed hydrogen gas pressures during the gasarite solidification of copper [20,22].

The use of a polynomial with five degrees in this work to predict the shapes of lotus-type pores in solids is sufficiently accurate compared with other polynomials of different degrees, as seen in Figure 3a. The predicted maximum diameter, length, and aspect ratio versus solidification rate are confirmed in accordance with the available experimental data provided by Park et al. [22] for the solidification of copper imposed by different hydrogen gas pressures at a top free surface, as shown in Figure 3b–d. The experimental data were selected for imposed hydrogen gases of 1 and 2 MPa and higher solidification rates of 20, 50, and 100 mm/min. Park et al. [22] used a continuous casting technique in a pressurized hydrogen atmosphere to observe the formation and growth of lotus-type pores in copper. The molten copper dissolving the hydrogen was pulled downward and solidified through a cooled mold at a given solidification rate. The apparatus chamber can be pressurized up to 3.0 MPa, whereas an up to 700 mm long solidified ingot slab can be produced. Pure copper (99.9 wt.% Cu) was melted in the crucible via radiofrequency induction heating at a temperature of around 1573 K. The other selected working variables are listed in Table 2 of a previous work [20]. An increase in either the solidification rate or the imposed hydrogen gas pressure was seen to decrease both the length and maximum diameter of lotus-type pores, which can be determined from Equations (9) and (20), respectively, or the shape of the presented lotus-type pores. For a detailed prediction, refer to the table provided in a previous work applying Henry’s law in nonmetals [21]. The present work was also favorably compared with numerical predictions, which involved the proposal and resolution of experimental measurements for isolated pore formation [19], and a set of simultaneous first-order ordinary differential equations [23]. The relatively constant aspect ratio during solidification contradicts the increase in the aspect ratio with the solidification rate suggested by Liu and Li [24]. The vertical lines in Figure 3b–d were drawn from the experimental results rather than from a computation. The theoretical results vary if the temperature- and concentration-dependent working parameters are allowed to change. In view of larger experimental uncertainties, the pore diameter and length of lotus pores with solidification rates less than 20 mm/min are not presented. A significant deviation between the predicted and measured maximum radius is not entirely clear but can be attributed to errors induced by the experimental measurements, stemming from challenges in maintaining a uniform and constant solidification rate, pressurizing the surrounding gas, and reliably capturing the shape of lotus-type pores, among other factors.

**Figure 4:**
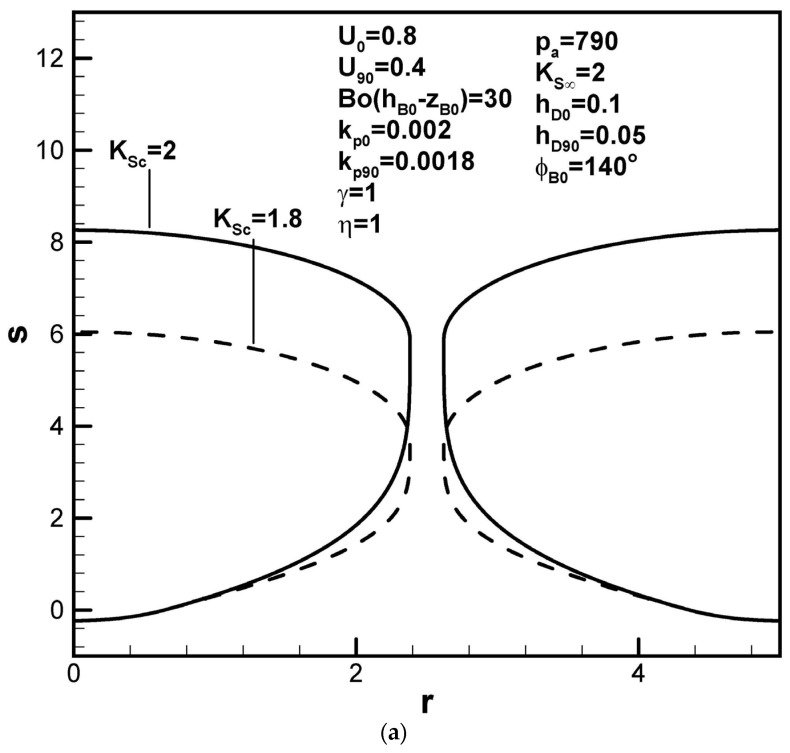

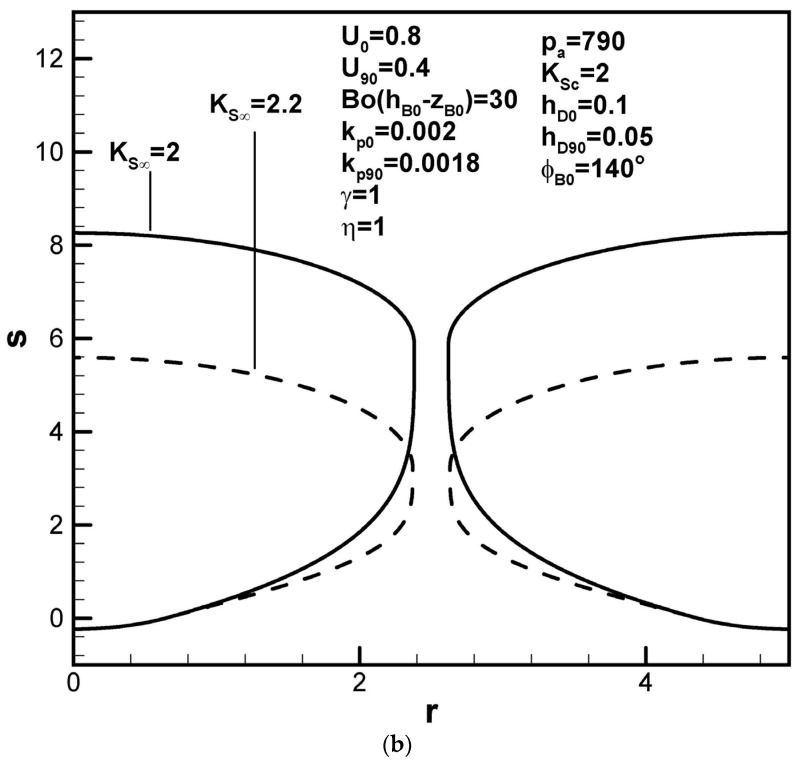
Predicted shapes of lotus-type pores for different dimensionless Sieverts’ law constants at the (**a**) bubble cap and (**b**) top free surface, subject to the given dimensionless working parameters.

All the following figures are presented in dimensionless forms, which are often adopted for general studies. Figure 4a shows that lotus-type pores become longer as the dimensionless Sieverts’ law constant at the cap increases. Since all the length scales are normalized by the initial apex radius, the shapes of the fusion zone are the same in the dimensionless and dimensional expressions. From the term $\left(\sqrt{p_{g90}}/K_{Sc}-\sqrt{\gamma p_{a}}/K_{S\infty }\right)$$F_{2}(r_{B90},h_{D90},U_{90})$ in Equation (9), the increase in Sieverts’ law constant at the cap can be seen to reduce the solute amount in the boundary layer on the cap at the maximum radius. Increasing Sieverts’ law constant at the top surface, however, decreases pore length, as presented in Figure 4b. This can be seen from term ($\sqrt{\gamma p_{a}}/K_{S\infty }$)$F_{4}(\phi_{B0},k_{p0},r_{B0},w,U_{0})$ in Equation (9), showing decreased solute content in the boundary layer on the solidification front at the initial time.

**Figure 5:**
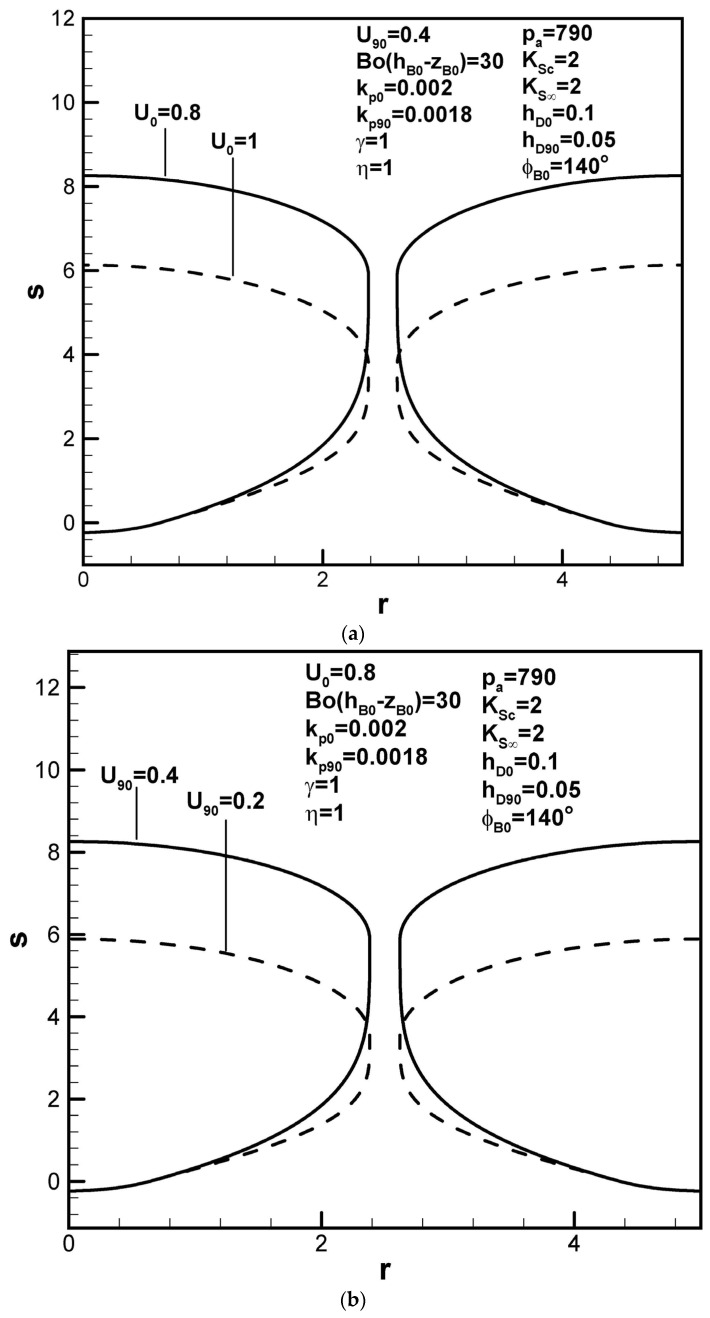
Predicted shapes of lotus-type pores for different dimensionless solidification rates at the (**a**) initial contact angles and (**b**) maximum radius, subject to the given dimensionless working parameters.

Increasing the solidification rate at the initial time shortens the pore length, as can be seen in Figure 5a, due to decreases in the boundary layer thickness and solute content in the boundary layer on the solidification front at the initial time (see Equation (9)). A higher dimensionless solidification rate has also been shown to readily entrap the bubble. From a dynamics viewpoint, the time scale of ${\tilde{R}}_{0}/\tilde{U}$ for the rapid solidification rate characterized by negligible solute transport is so small that the solute gas pressure in the pore maintains a high value. The smaller pore size thus looks like a sphere for a higher solidification rate at the initial time. The dimensionless pore length, however, increases with the dimensionless solidification rate at the maximum radius, as shown in Figure 5b. This can be interpreted by decreases in the boundary layer thickness and solute amount in the boundary layer on the solidification front at a contact angle of 90 degrees.

**Figure 6:**
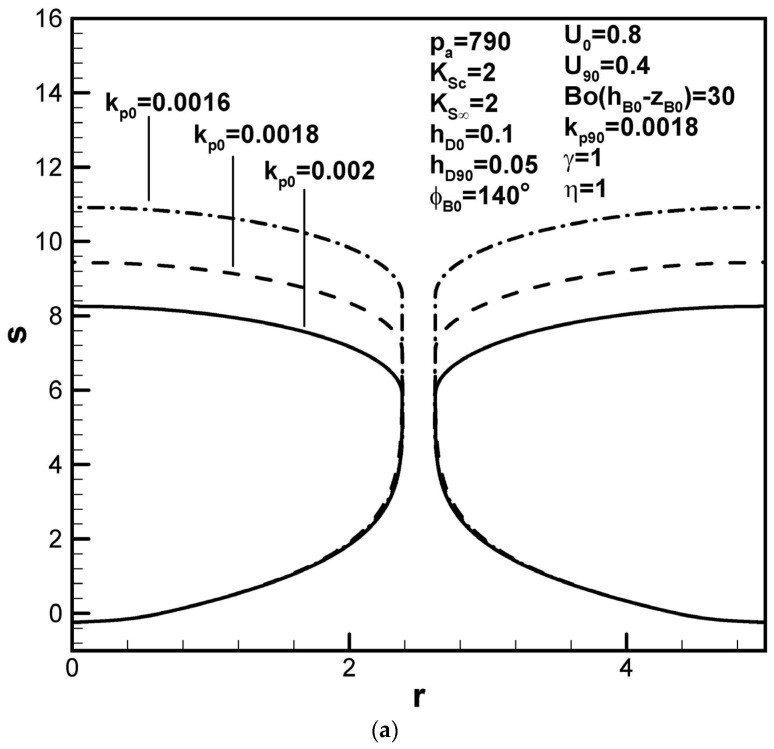

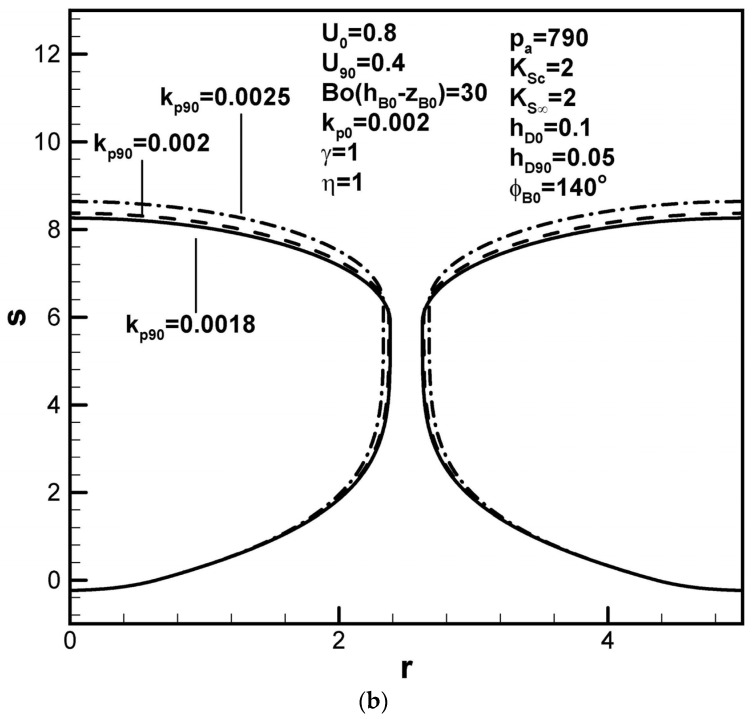
Predicted shapes of lotus-type pores for different partition coefficients at the (**a**) initial contact angles and (**b**) maximum radius, subject to the given dimensionless working parameters.

**Figure 7:**
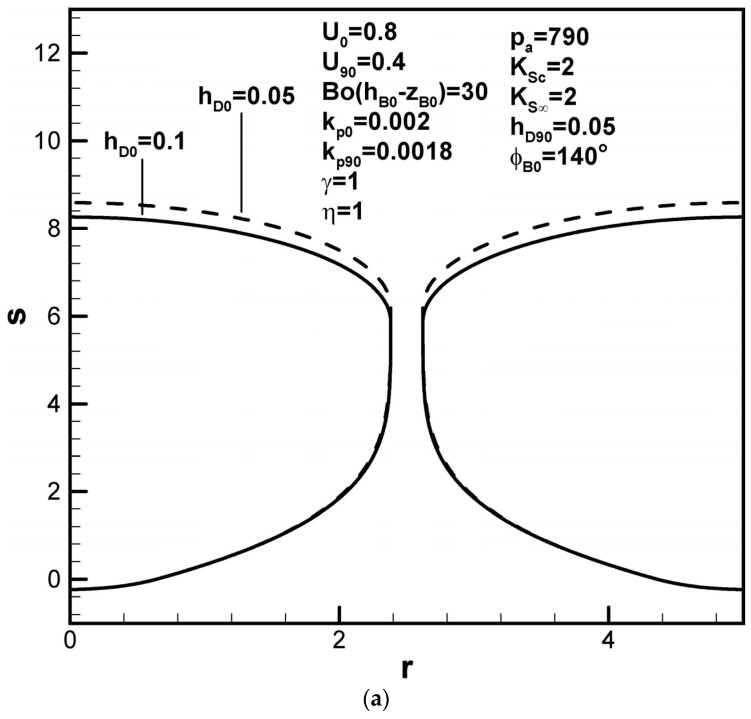

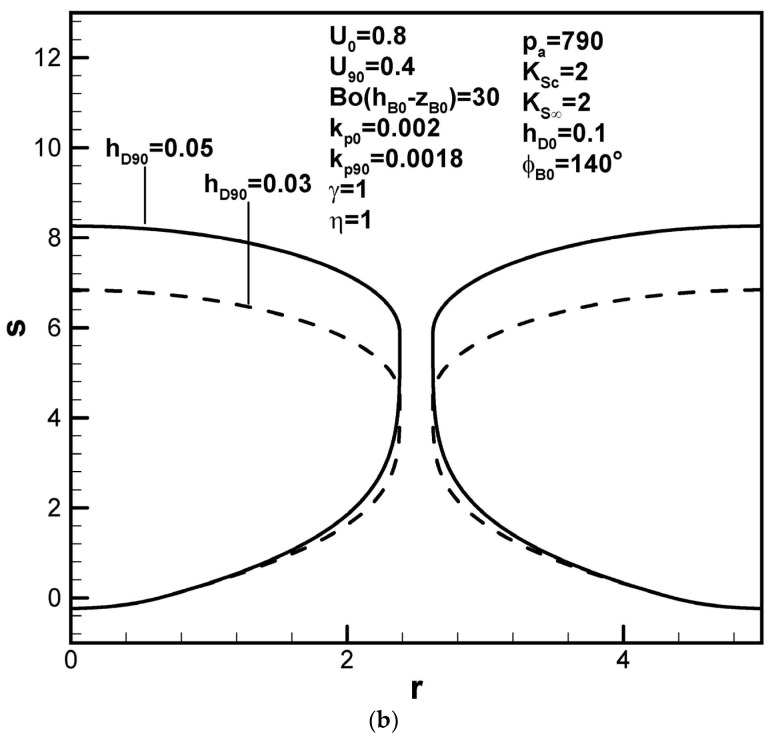
Predicted shapes of lotus-type pores for different dimensionless mass transfer coefficients at the (**a**) initial contact angles and (**b**) maximum radius, subject to the given dimensionless working parameters.

Dimensionless pore length increases as the partition coefficient at the initial time decreases, while that at the maximum radius increases, as shown in Figure 6a,b, respectively. They are consequences of an increase and a decrease in the solute content in the boundary layer on the solidification front found from functions $F_{4}(\phi_{B0},k_{p0},r_{B0},w,U_{0})$ and $F_{5}(k_{p90},r_{B90},w,U_{90})$, respectively. In view of the increase in the solute amount in the boundary layer on the cap at the initial time, a decrease in the dimensionless mass transfer coefficient at the initial time increases the dimensionless pore length, as shown in Figure 7a. On the other hand, pore length increases with the dimensionless mass transfer coefficient at the cap at the maximum radius, as shown in Figure 7b.

**Figure 8:**
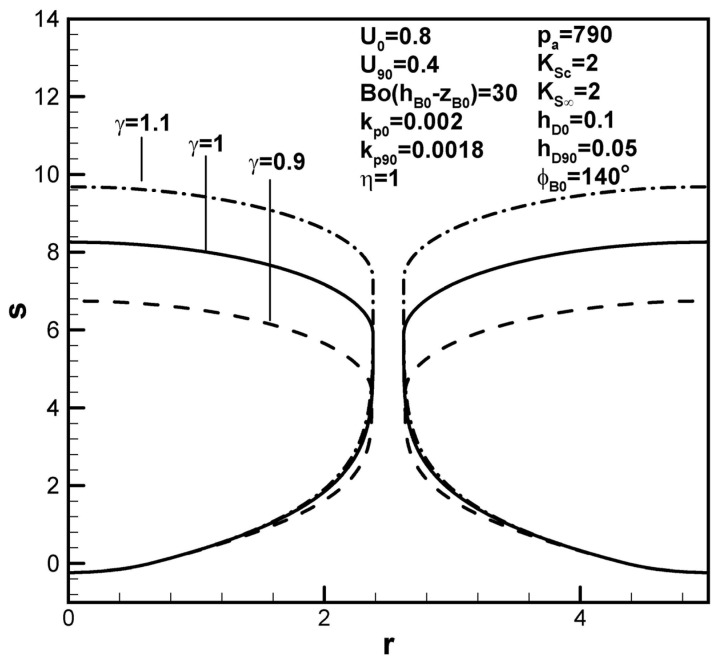
Predicted shapes of lotus-type pores for different mole fractions of the solute gas at the top surface, subject to the given dimensionless working parameters.

**Figure 9:**
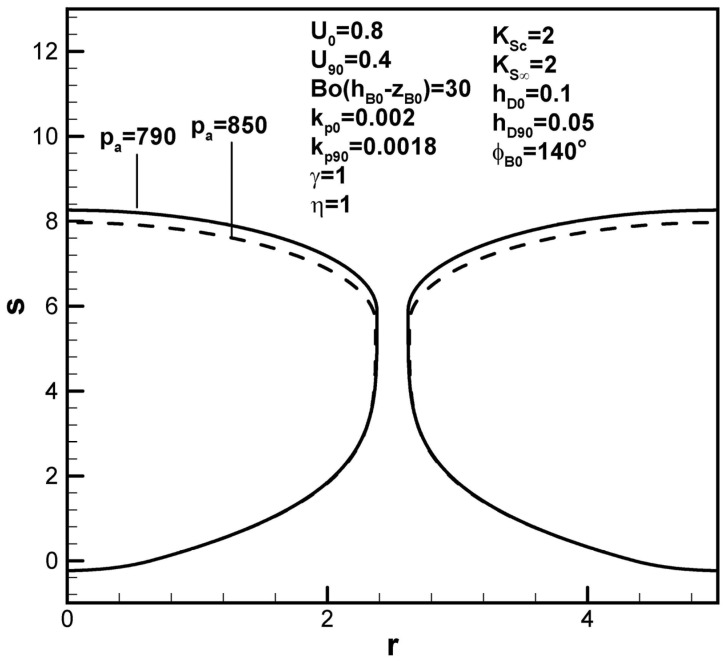
Predicted shapes of lotus-type pores for different dimensionless ambient pressures, subject to the given dimensionless working parameters.

**Figure 10:**
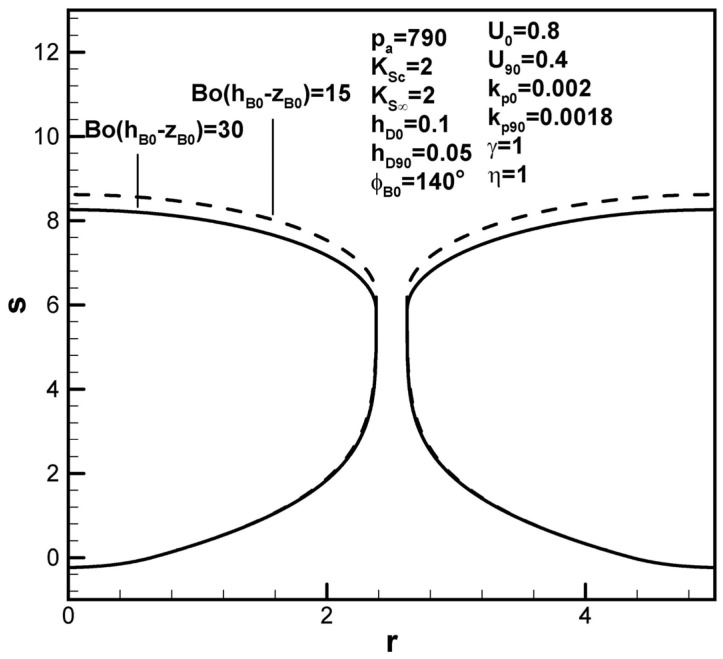
Predicted shapes of lotus-type pores for different dimensionless hydrostatic pressures, subject to the given dimensionless working parameters.

Dimensionless pore length is found to increase with solute concentration in the ambient gas on the top surface, as shown in Figure 8, interpreted to be due to the increase in the solute amount in the boundary layer on the solidification front at the initial time. Referring to Equations (13) and (14), the function $F_{4}(\phi_{B0},k_{p0},r_{B0},w,U_{0})$ is generally greater than $F_{5}(k_{p90},r_{B90},w,U_{90})$. Contrary to previous work [19], Figure 9 shows that dimensionless pore length increases as the dimensionless ambient pressure on the top surface decreases. The effects of ambient pressure on the top surface on the pore length can vary (see Equation (9)). A decrease in the dimensionless ambient pressure can reduce the solute content in the pore as well as in the boundary layer on the cap and solidification front at the initial time and maximum radius (see Equation (9)). The major factor in this case is attributed to decreases in solute gas pressure in the pore and concentration boundary layer on the solidification front at the maximum radius. In view of the increase in the solute amount in the boundary layer on the cap at the maximum radius, an increase in the dimensionless hydrostatic pressure decreases the dimensionless pore length, as shown in Figure 10.

**Figure 11:**
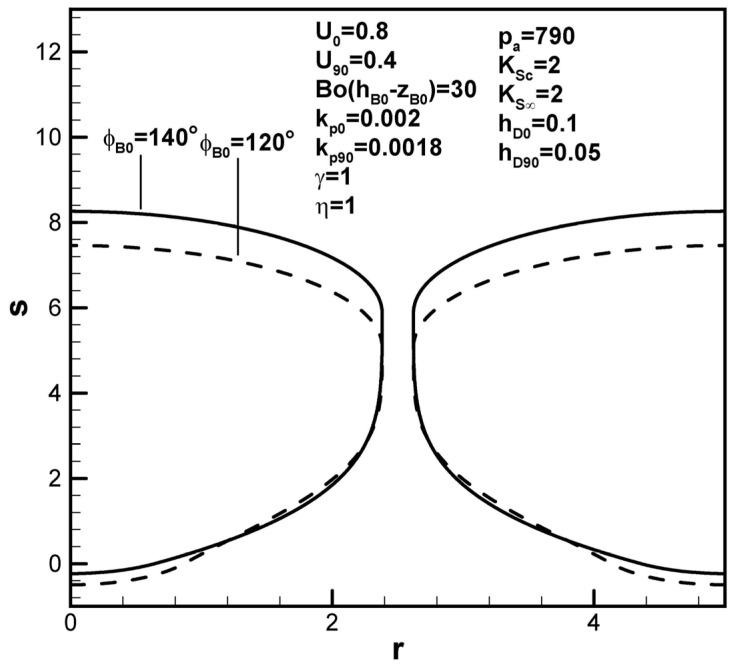
Predicted shapes of lotus-type pores for different initial contact angles, subject to the given dimensionless working parameters.

**Figure 12:**
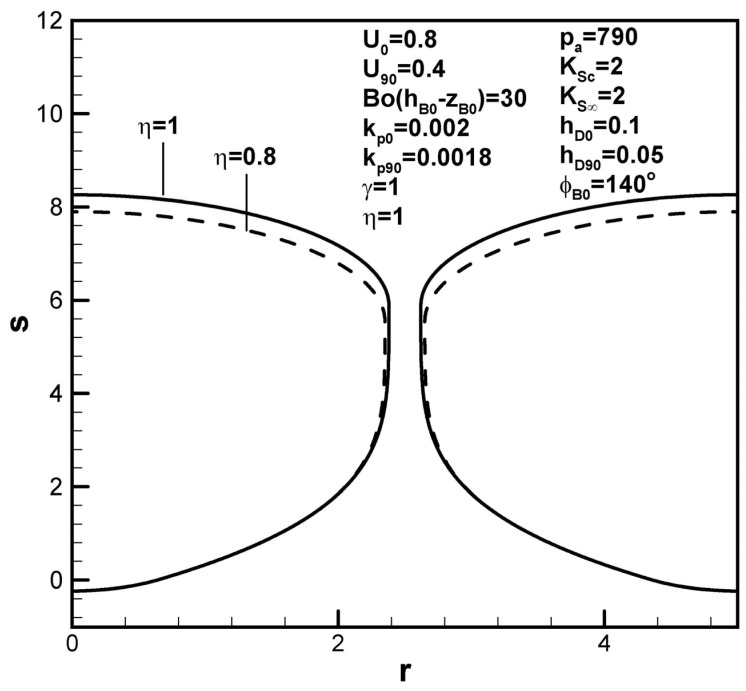
Predicted shapes of lotus-type pores for different solute transport parameters, subject to the given dimensionless working parameters.

**Figure 13:**
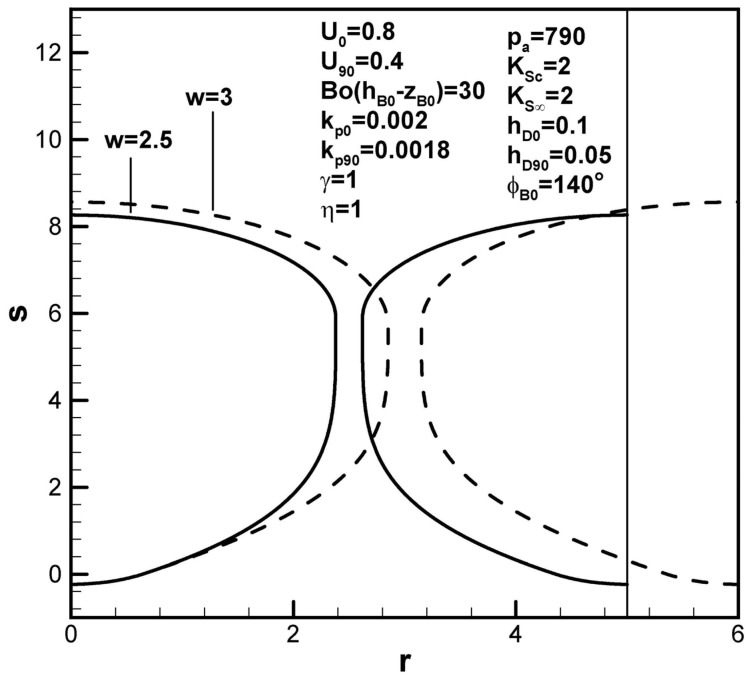
Predicted shapes of lotus-type pores for different dimensionless inter-pore spacings, subject to the given dimensionless working parameters.

Figure 11 shows that dimensionless pore length increases with the initial contact angle, resulting from an increase in the solute amount in the boundary layer on the solidification front at the initial time. Dimensionless pore length increases with the solute transport parameter, as shown in Figure 12 because a decrease in the solute content in the boundary layer on the solidification front increases the solute amount in the pore and pore radius (see Equation (19)). The solute gas pressure in the pore at the maximum radius thus decreases (see Equation (17)). The dimensionless length of lotus-type pores therefore increases as the solute gas pressure in the pores decreases at the maximum radius (see Equation (9)). Figure 13 shows that dimensionless pore length increases with dimensionless inter-pore spacing, attributed to an increase in the solute amount in the boundary layer on the solidification front at the initial time.

## 4. Conclusions

This study extends previous models of lotus-type pores from nonmetals to metals, incorporating total solute conservation in the pores, boundary layers on the caps and solidification fronts, and different physico-chemical equilibrium conditions and segregation at the gas–liquid and solid–liquid interfaces. The following conclusions were drawn:

1. The morphology of lotus-type pores was parametrically predicted using general algebraic equations. The predicted shapes of the lotus-type pores align with Park et al.’s experimental results for copper solidified with dissolved hydrogen.
2. The increase in dimensionless pore length with Sieverts’ law constant at the cap results from a decrease in the solute amount in the boundary layer on the cap at the maximum radius. Conversely, as Sieverts’ law at the free surface decreases, the pore length increases due to an initial increase in the solute content in the boundary layer on the solidification front.
3. Dimensionless pore length increases as the mass transfer coefficients at the initial time decrease, whereas that at the maximum radius increases. They are attributed to an increase and decrease in solute content in the boundary layer on the cap at the initial time and maximum radius, respectively.
4. Dimensionless pore length increases as the solidification rate at the initial time decreases while that at the maximum radius increases. They are attributed to an increase and decrease in solute amount in the boundary layer on the solidification front at the initial time and maximum radius, respectively. For the same reason, dimensionless pore length increases as the partition coefficient at the initial contact angle decreases, whereas that at the maximum radius increases.
5. The effects of ambient pressure on pore length can vary. In this study, the increase in dimensionless pore length due to a decrease in ambient pressure on the top free surface can be attributed to a primary decrease in the solute amount in the boundary layer on the solidification front at the maximum radius. Dimensionless pore length increases as hydrostatic pressure decreases, resulting from a decrease in the solute amount in the boundary layer on the cap at the maximum radius.
6. Dimensionless pore length increases with the initial contact angle, solute gas concentration on the top free surface, and inter-pore spacing due to an increase in solute amount in the boundary layer on the solidification front at the initial time. On the other hand, the increase in dimensionless pore length with the solute transport parameter is due to a decrease in solute content in the boundary layer on the solidification front at the maximum radius.

## Data Availability

Data is contained within the article. The original contributions presented in the study are included in the article, further inquiries can be directed to the corresponding author.

## Nomenclature

*A*	cap surface area, $A=\tilde{A}/{\tilde{R}}_{0}^{2}$
*A*, *B*, …, *G*	coefficient functions
*a*, *b*, …, *f*	coefficients
*Bo*	Bond number, $Bo\equiv {\tilde{\rho }}_{l}g{\tilde{R}}_{0}^{2}/\sigma$
C	initial solute concentration, $C=\tilde{C}{\tilde{R}}_{u}{\tilde{T}}_{m}/{\tilde{\rho }}_{l}g{\tilde{R}}_{0}$
*D*	solute diffusivity
$F_{1},F_{2},\ldots ,F_{5}$	coefficient functions
$h_{B}$	liquid depth
$h_{D}$	mass transfer coefficient, $h_{D}={\tilde{h}}_{D}{\tilde{R}}_{0}/{\tilde{D}}_{l}$
$k_{p}$	partition coefficient
$K_{S}$	Sieverts’ law constant, $K_{S}={\tilde{K}}_{S}\sqrt{\sigma /{\tilde{R}}_{0}}/{\tilde{R}}_{u}{\tilde{T}}_{m}$
l	length
*p*	pressure, $p\equiv \tilde{p}{\tilde{R}}_{0}/\sigma$
*r*	coordinate, $r\equiv \tilde{r}/{\tilde{R}}_{0}$
*R*	apex radius, $R\equiv \tilde{R}/{\tilde{R}}_{0}$
$R_{u}$	gas constant
*s*	solidification front location
*t*	time, $t=\tilde{t}\tilde{D}/{\tilde{R}}_{0}^{2}$
*T*	temperature
*U*	solidification rate, *U* = *ds*/*dt*
w	inter-pore spacing
*z*	coordinate, $z\equiv \tilde{z}/{\tilde{R}}_{0}$
Greek letter	
σ	surface tension
γ	mole fraction of solute gas
ρ	density
δ	concentration boundary layer thickness
η	solute transport parameter
ϕ	contact angle
Subscripts	
*a*	ambient
*B*	base
*c*	cap
*cr*	intersection angle between concentration boundary layers
*g*	gas
l	liquid
*m*	melting temperature
*s*	solid
*S*	Sieverts’ law
0	initial state
90	90 degrees
∞	far from solidification front
Superscripts	
~	dimensional quantity
